# Simultaneously improving accuracy and computational cost under parametric constraints in materials property prediction tasks

**DOI:** 10.1186/s13321-024-00811-6

**Published:** 2024-02-16

**Authors:** Vishu Gupta, Youjia Li, Alec Peltekian, Muhammed Nur Talha Kilic, Wei-keng Liao, Alok Choudhary, Ankit Agrawal

**Affiliations:** 1 Department of Electrical and Computer Engineering, Northwestern University, Evanston, USA; 2 Department of Computer Science, Northwestern University, Evanston, USA

**Keywords:** Deep Learning, Materials Informatics, Predictive Modeling

## Abstract

Modern data mining techniques using machine learning (ML) and deep learning (DL) algorithms have been shown to excel in the regression-based task of materials property prediction using various materials representations. In an attempt to improve the predictive performance of the deep neural network model, researchers have tried to add more layers as well as develop new architectural components to create sophisticated and deep neural network models that can aid in the training process and improve the predictive ability of the final model. However, usually, these modifications require a lot of computational resources, thereby further increasing the already large model training time, which is often not feasible, thereby limiting usage for most researchers. In this paper, we study and propose a deep neural network framework for regression-based problems comprising of fully connected layers that can work with any numerical vector-based materials representations as model input. We present a novel deep regression neural network, iBRNet, with branched skip connections and multiple schedulers, which can reduce the number of parameters used to construct the model, improve the accuracy, and decrease the training time of the predictive model. We perform the model training using composition-based numerical vectors representing the elemental fractions of the respective materials and compare their performance against other traditional ML and several known DL architectures. Using multiple datasets with varying data sizes for training and testing, We show that the proposed iBRNet models outperform the state-of-the-art ML and DL models for all data sizes. We also show that the branched structure and usage of multiple schedulers lead to fewer parameters and faster model training time with better convergence than other neural networks. Scientific contribution: The combination of multiple callback functions in deep neural networks minimizes training time and maximizes accuracy in a controlled computational environment with parametric constraints for the task of materials property prediction.

## Introduction

One of the most critical aspects of modern computational materials science is to perform accurate materials property prediction to, in turn, discover new materials with desirable characteristics from the near-infinite materials space. To achieve this goal, researchers have applied machine learning (ML) and deep learning (DL) algorithms to large-scale datasets derived through experiments and high throughput simulations such as density functional theory (DFT) calculations [1–5] to understand materials better and predict their properties [6–10] leading to the novel paradigm of materials informatics [11–19]. Materials property prediction is generally a regression-based task where various types of numerical features derived from domain knowledge, such as composition-based and structure-based features, are used as input to train and generate a predictive model [20–25]. Since the materials are represented in the form of a one-dimensional numerical vector, traditional ML algorithms such as Random Forest and Support Vector Machines and neural networks based deep learning (DL) models composed of fully connected layers are widely used to perform the regression task [26–31].

In an attempt to obtain a highly accurate predictive model for the regression-based task of materials property prediction, researchers have proposed deep learning models with complex input types, network components, and architecture design [32–41]. Work in [34] used a 17-layered deep neural network composed of fully connected layers with varying layer sizes called ElemNet which automatically captures the essential chemistry between the elements of a compound using elemental fractions without any domain knowledge based feature engineering as input to predict the formation enthalpy of materials. ElemNet was applied in [37], where they applied a transfer learning technique from a large DFT dataset to an experimental dataset to improve the accuracy of the predictive model trained on experimental formation enthalpy. Work in [40, 41] proposed deep-learning framework based on branched residual learning with fully connected layers called BRNet which can efficiently build accurate models for predicting materials properties with fewer parameters and faster model training time. Zhou et al. [42] used a neural network with a single fully connected layer to predict formation energy from high-dimensional vectors learned from Atom2Vec. Work in [32] used a continuous filter convolutional neural network called SchNet to model quantum interactions in molecules for the interatomic forces and total energy. SchNet was extended in [33] where they added an edge update network to allow for neural message passing between atoms for better predictions of molecular and materials properties. Crystal graph convolution neural network (CGCNN) proposed in [35] provides a universal and interpretable representation of crystalline materials by directly learning material properties from the connection of atoms in the crystal. CGCNN was improved in [36] where they incorporated Voronoi tessellated crystal structure information, optimized chemical representation of interatomic bonds in the crystal graph, and used explicit 3-body correlations of neighboring constituent atoms. Work in [43] developed a universal MatErials Graph Network (MEGNet) model with global state attributes for materials property prediction of molecules and crystals. Goodall and Lee developed Roost [38] that combines the stoichiometry of a compound with an atom-based embedding using a message-passing neural network comprised of dense weighted graphs to improve the predictive ability. Recently, Choudhary and DeCost developed Atomistic Line Graph Neural Network (ALIGNN) [39], which combines angular information along with the existing atom and bond information to obtain high accuracy models for improved materials property prediction.

In general, most of the pre-existing works focus on using complex network components, input types, and architecture design to improve the predictive ability of the trained model, thereby making a trade-off between model accuracy with computational resources and training time. However, it can be challenging to leverage such complex components to build predictive models as these changes require higher computational resources and training time. Moreover, these complex architectures use little to no callback functions, such as early stopping and learning rate schedulers, during their training process to help generalize and improve the performance of the trained model, even though various applications have been shown to benefit from the use of it [44, 45], thereby possibly requiring more rigorous random hyperparameter optimization in an attempt to obtain an accurate model for a specific materials property. Hence, in this work, we focus on the problem of building an effective and efficient deep neural network architecture with higher accuracy that has a lower computational cost during model training in a controlled computational environment (17-layers in our case) rather than introducing complex network components, input types, and architecture design to try and boost model performance as done in recent works [35, 36, 38–41, 43, 46]. For this purpose, we propose and analyze a deep learning framework composed of deep neural networks and multiple callback functions that has less computational cost and higher accuracy and can be used to predict materials properties using tabular representations. Since we encounter a lot of regression-based problems in physical sciences, and the datasets used to create a model consist of tabular data, the model architectures are mainly composed of fully connected layers. However, learning the regression mapping from input to output using fully connected layers is comparatively more challenging than the classification problem due to its highly non-linear nature. Hence, to simultaneously minimize training time and maximize accuracy in a controlled computational environment with parametric constraints, we propose a novel approach based on a combination of multiple callback functions and a deep neural network composed of fully connected layers.

The proposed approach leverages multiple callback functions in a deep neural network, building upon a pre-existing 17-layered deep neural network branched residual network (BRNet) as the base architecture, which comprises of a series of stacks, each composed of a fully connected layer and LeakyReLU [47] with a branched structure in the initial layers and residual connections after each stack for better convergence during the training. For simplicity, we call our proposed model as improved branched residual network (iBRNet). We compare iBRNet against multiple baseline deep regression networks (all of which are made using 17 layers, with each layer comprised of the same number of neurons): ElemNet with fully connected layers and dropout at variable intervals of the architecture, individual residual network (IRNet) with fully connected layers, batch normalization, and residual connections after each layer, branched network (BNet) with fully connected layers and branching at the initial layers of the architecture, and branched residual network (BRNet) with fully connected layers, branching at the initial layers of the architecture and residual connection after each layer. We also compare iBRNet against other well-known deep neural networks [48–50] that use composition-based features as model input. We focus on the design problem of predicting the formation enthalpy of inorganic materials from a tabular input vector composed of 86 features representing composition-based elemental fractions from the Open Quantum Materials Database (OQMD) [3], Automatic Flow of Materials Discovery Library (AFLOWLIB) [51], Materials Project (MP) [4], and Joint Automated Repository for Various Integrated Simulations (JARVIS). We also evaluated the performance of the iBRNet using other materials properties in OQMD, AFLOWLIB, MP, and JARVIS datasets and found that iBRNet consistently outperforms the networks trained in a controlled computational environment with parametric constraints on the prediction tasks. We also observe that the use of multiple callback functions during the training phase of a deep neural network leads to significantly faster convergence than existing approaches that use little to no callback functions in their training phase. iBRNet leverages an intuitive and straightforward approach of leveraging multiple callback functions during the training phase of a deep neural network without requiring any additional modification to the architecture or domain-dependent model engineering, thereby making it easy and useful for researchers working not only on materials science but other scientific domains to train a predictive model for their regression-based tasks.

## Results and discussion

### Datasets

We use four datasets of DFT-computed properties in this work: Open Quantum Materials Database (OQMD) [3], Automatic Flow of Materials Discovery Library (AFLOWLIB) [51], Materials Project (MP) [4], Joint Automated Repository for Various Integrated Simulations (JARVIS) [5]. We only keep the most stable structure available in the database to deal with duplicates arising due to different structures of the same composition, i.e., each data entry corresponds to the lowest formation energy among all compounds with the same composition, representing its most stable crystal structure. Detailed descriptions of the datasets used to evaluate our methods are shown in Table 1.

**Table 1:** Datasets used in this work

Dataset	Data size	No. of properties
OQMD [3]	345,134	3
AFLOWLIB [51]	234,299	3
MP [4]	89,181	5
JARVIS [5]	19,994	5

OQMD, AFLOWLIB, MP, and JARVIS were downloaded from the website of the databases, whereas all the other datasets were obtained using Matminer [52]. For evaluation, all the datasets are randomly split with a fixed random seed and stratification based on the number of elements in a compound (to make the model train, validate, and test on the same proportion of compound with variable no. of elements) into training, validation, and test sets in the ratio of 81:9:10.

### Model architecture design

We use BRNet [40, 41] as our base architecture as it was shown to perform better than traditional machine learning models and other existing neural networks with the same parametric constraints. A detailed explanation of the model architectures used in this work is provided in the Methods section. To improve the performance of the existing BRNet model without introducing additional computational parameters, we made some changes to the components and evaluated how it affects its accuracy and training time for the task of predicting formation energy using training data from OQMD, AFLOWLIB, MP, and JARVIS.

The BRNet is modified by combining “reduce learning rate on plateau (RLROP)” with “early stopping (ES)” callback functions. ES is used to stop the model training if the validation loss does not improve after a certain number of specified epochs and save the model with the best validation error to prevent the model from overfitting. RLROP is used to reduce the learning rate by a factor (generally between two-ten) if the validation loss stops improving after a certain number of specified epochs to help the model get out of the learning stagnation state. These callback functions are often seen used in deep neural networks composed of simpler neural networks, such as a fully connected network but rarely seen in more advanced neural networks, such as graph neural networks, possibly requiring more rigorous random hyperparameter optimization in an attempt to obtain an accurate model for a specific materials property. Next, we perform model training using different combinations of epochs required to activate the callback functions used in the iBRNet (ES and RLROP) to see the effect on the accuracy and training time of the model. We start with a combination of 5/10 epochs for RLROP/ES and go till 95/100 epochs (e.g. of combinations: 5/10, 10/15,...95/100) where the difference in the number of epochs between the two callback functions is set to five for generalizability. For RLROP, we change the learning rate by a factor of 10 from $1 \times 10^{-4}$ to $1 \times 10^{-8}$ as the model stops improving.

**Table 2:** Validation MAE and training time for different combinations of RLROP/ES on OQMD, AFLOWLIB, MP, and JARVIS

RLROP/ES Combination	OQMD		AFLOWLIB		MP		JARVIS	
	Validation MAE	Training Time (s)	Validation MAE	Training Time (s)	Validation MAE	Training Time (s)	Validation MAE	Training Time (s)
5/10	0.041	8477	0.045	5542	0.106	1216	0.073	339
10/15	0.040	10003	0.043	8424	0.105	1533	0.068	602
15/20	0.040	14779	0.043	8056	0.104	2137	0.065	926
20/25	0.038	20389	0.043	12870	0.103	2623	0.065	965
25/30	0.039	18192	0.043	12593	0.102	3806	0.065	1005
30/35	0.039	24020	0.043	15289	0.100	4597	0.065	1231
35/40	0.038	30933	0.043	17979	0.102	6053	0.065	1000
40/45	0.039	28640	0.043	18908	0.103	10103	0.064	1534
45/50	0.038	38252	0.043	19255	0.101	5514	0.065	1828
50/55	0.038	28982	0.043	22525	0.100	9021	0.065	1619
55/60	0.038	33719	0.043	23637	0.101	7860	0.066	1750
60/65	0.038	34438	0.043	21431	0.100	10317	0.065	1915
65/70	0.038	44597	0.043	23908	0.101	10866	0.064	2265
70/75	0.038	53111	0.043	25401	0.099	8753	0.065	1817
75/80	0.038	45250	0.043	27630	0.100	8161	0.066	2106
80/85	0.039	72469	0.043	26827	0.099	8624	0.065	3564
85/90	0.038	56431	0.043	32344	0.099	11587	0.064	2363
90/95	0.038	65028	0.043	34705	0.099	10913	0.065	3730
95/100	0.038	79660	0.043	46944	0.098	10642	0.065	2363

Table 2 shows the validation MAE and training time for different combinations of RLROP/ES. From Table 2 we can see that initially, the validation MAE decreases as we increase the number of epochs required to activate the RLROP and ES callback functions. Then we see a stagnation in the validation MAE of the prediction task for all four datasets used in the analysis. Also, even though the validation MAE does not decrease after a certain combination of RLROP/ES, we observe a constant increase in the training time as we increase the number of epochs required to activate the RLROP and ES callback functions. Hence, we narrow down the RLROP/ES combinations used for performing model training for iBRNet 45–50 only to perform model testing on the holdout test set to have a fair comparison with other models with parametric constraints for the rest of the analysis. Next, we compare the performance of our proposed model against its base architecture as well as other DL models with the same parametric constraint, on the holdout test set.

**Table 3:** Test MAE and training time of different models for prediction task of “Model Architecture Design”

Dataset	Evaluation	Models				
(Size)	Metrics	ElemNet	IRNet	BNet	BRNet	iBRNet
OQMD	MAE	0.0491	0.0421	0.0423	0.0409	0.0372
(345,134)	(Training Time (s))	(70851)	(258367)	(97587)	(106658)	(38252)
AFLOWLIB	MAE	0.0581	0.0508	0.0481	0.0468	0.0433
(234,299)	(Training Time (s))	(16493)	(94373)	(18123)	(30501)	(19255)
MP	MAE	0.1210	0.1166	0.1118	0.1063	0.1035
(89,181)	(Training Time (s))	(5951)	(25249)	(8367)	(15677)	(5514)
JARVIS	MAE	0.0829	0.0942	0.0713	0.0705	0.0664
(19,994)	(Training Time (s))	(2036)	(3547)	(1913)	(4467)	(1828)

Table 3 shows that the proposed model significantly outperforms the existing deep neural network architectures, which do not use multiple callback functions for model training, on the prediction task for all the datasets. We also observed that multiple callback functions significantly reduce the training time without changing the number of parameters used to construct the architecture, which illustrates its benefit over ElemNet, IRNet, BNet, and BRNet for the design task. Moreover, the difference in test MAE and training time between BRNet and iBRNet is also significant, suggesting that simply introducing a meaningful set of callback functions can help improve the performance of the deep neural network architectures trained in a controlled computational environment with the same parametric constraint. Additionally, we observe that the MAE of the trained model does not always decrease with the increase in the number of data points, like in the case of the model trained using the MP dataset, which shows higher model error as compared to the model trained using the JARVIS dataset. It would be interesting to see if it is possible to analyze the underlying cause of this by exploring the parametric settings of the DFT simulations used to generate the MP dataset.

### Other materials properties

Next, we analyze the performance of our proposed model for predicting materials properties other than formation enthalpy. To show the impact on the performance, we compare the performance of our proposed network against DL networks that do not incorporate multiple callback functions for their model training.

**Table 4:** Test MAE and training time of different models for each of the materials properties for the prediction task of “Other materials properties”

Dataset	Property	Evaluation	Models				
	(Size)	Metrics	ElemNet	IRNet	BNet	BRNet	iBRNet
OQMD	Band Gap	MAE	0.0517	0.0544	0.0500	0.0476	0.0449
	(345,134)	(Training Time (s))	(27428)	(119341)	(37516)	(37914)	(29021)
	Stability	MAE	0.0509	0.0470	0.0447	0.0429	0.0414
	(345,134)	(Training Time (s))	(41206)	(131252)	(62139)	(93733)	(23872)
AFLOWLIB	Density	MAE	0.2271	0.1855	0.1844	0.1762	0.1656
	(234,299)	(Training Time (s))	(8160)	(81221)	(22021)	(23989)	(20849)
	EGap	MAE	0.1446	0.1398	0.1161	0.1078	0.0940
	(14,751)	(Training Time (s))	(628)	(2651)	(642)	(866)	(861)
MP	Band Gap	MAE	0.3417	0.3159	0.3170	0.3151	0.2943
	(89,181)	(Training Time (s))	(5780)	(18998)	(9458)	(9346)	(4493)
	Density	MAE	0.3732	0.3734	0.3489	0.3441	0.3293
	(89,181)	(Training Time (s))	(11080)	(25609)	(12724)	(13433)	(6260)
	E Above Hull	MAE	0.1080	0.1120	0.1031	0.1086	0.1005
	(89,181)	(Training Time (s))	(2461)	(6763)	(5790)	(10339)	(4993)
	Total Magnetization	MAE	1.4277	1.3947	1.4123	1.4331	1.4094
	(89,181)	(Training Time (s))	(2095)	(6394)	(2369)	(2916)	(2317)
JARVIS	Gap OPT	MAE	0.2935	0.2995	0.2645	0.2602	0.2451
	(17,924)	(Training Time (s))	(1200)	(2561)	(1042)	(1113)	(1082)
	Bulk Modulus	MAE	11.556	11.707	11.791	10.632	10.115
	(8,199)	(Training Time (s))	(792)	(1270)	(427)	(879)	(561)
	Shear Modulus	MAE	10.640	10.754	11.101	9.9359	9.6818
	(8,199)	(Training Time (s))	(585)	(764)	(274)	(527)	(397)
	Gap TBMBJ	MAE	0.5441	0.5259	0.4833	0.4967	0.4828
	(5,287)	(Training Time (s))	(373)	(792)	(257)	(276)	(241)

**Fig. 1:**
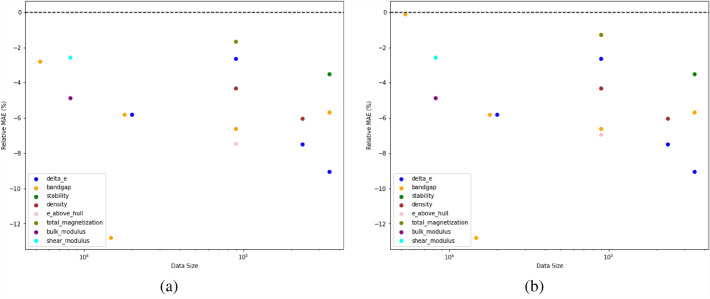
The figure indicates the percentage change in test MAE of the proposed iBRNet w.r.t (**a**) BRNet, and (**b**) best performing pre-existing model. The x-axis shows the dataset size on a log scale, and the y-axis shows the percentage change in test MAE from all the model training performed in Tables 3, 4 calculated as ((MAE_iBRNet_/MAE_Other_)–1) x 100$\%$

**Fig. 2:**
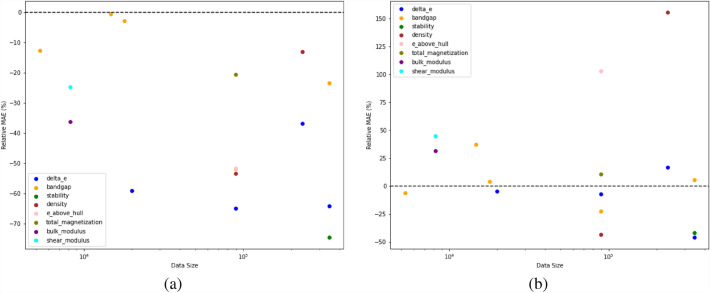
The figure indicates the percentage change in training time of the proposed iBRNet w.r.t (**a**) BRNet, and (**b**) best performing pre-existing model. The x-axis shows the dataset size on a log scale, and the y-axis shows the percentage change in training time from all the model training performed in Tables 3, 4 calculated as ((Time_iBRNet_/Time_Other_)–1) x 100$\%$

Table 4 shows that the proposed model with multiple callback functions always outperforms other DL models that do not incorporate multiple callback functions for their model training in terms of accuracy and training time. The performance of ElemNet and IRNet is almost always the worst, with ElemNet showing low accuracy and IRNet showing large training time, except for some cases where there are fewer data points for model training. We also observe that the training time of iBRNet is almost always faster as compared to its base architecture BRNet. iBRNet also shows better or comparable training time as compared to other architectures while keeping the best accuracy among all the models.This shows that a deep neural network significantly benefits from the use of multiple callback functions both in terms of improving accuracy and decreasing the training time. Similar to the previous observation, the MAE of the trained model does not always decrease with the increase in the number of data points for other materials properties like Band Gap as well. We also plot the percentage change in test MAE and training time of the proposed iBRNet against BRnet and best performing pre-existing model in Figs. 1,  2 respectively.

Figures 1, 2 show that iBRNet outperforms the existing DL models for most of the cases with up to 13% reduction in test MAE and 51% reduction in training time with BRNet as well as other pre-existing DL models which uses the same number of layers in the architecture for almost all materials properties in the four datasets used in the analysis. Although for some of the cases, pre-existing DL models (mostly ElemNet) have faster training time as compared to iBRNet, the test MAE of those pre-existing DL models is far worse as compared to iBRNet, making those DL models not very useful for further analysis. This clearly illustrates the benefit of incorporating multiple callback functions for traning deep neural networks.

### Other materials representation

Next, we investigate the adaptability of the proposed network by training models on an other materials representation as model input. Here, we train all the DL networks using a vector composed of 145 features representing composition-based physical attributes [21] for model input instead of 86 vector elemental fractions (EF) [34].

**Table 5:** Test MAE and training time of different models for each of the materials properties for prediction task of “Other materials representation”

Dataset	Property	Evaluation	Models				
	(Size)	Metrics	ElemNet	IRNet	BNet	BRNet	iBRNet
OQMD	Formation Energy/Atom	MAE	0.0841	0.0620	0.0527	0.0513	0.0478
	(345,134)	(Training Time (s))	(17177)	(117742)	(60088)	(95614)	(43220)
	Band Gap	MAE	0.0645	0.0503	0.0491	0.0512	0.0474
	(345,134)	(Training Time (s))	(18683)	(130030)	(40658)	(31271)	(30125)
	Stability	MAE	0.0725	0.0642	0.0526	0.0529	0.0497
	(345,134)	(Training Time (s))	(21556)	(87608)	(60030)	(53959)	(37015)
AFLOWLIB	Formation Energy/Atom	MAE	0.0688	0.0612	0.0501	0.0507	0.0463
	(234,299)	(Training Time (s))	(18437)	(48912)	(42212)	(30582)	(21103)
	Density	MAE	0.2263	0.1871	0.1884	0.1862	0.1746
	(234,299)	(Training Time (s))	(21416)	(77124)	(22172)	(34066)	(14534)
	EGap	MAE	0.1546	0.1140	0.1085	0.1144	0.1064
	(14,751)	(Training Time (s))	(781)	(5278)	(1500)	(1640)	(1620)
MP	Formation Energy/Atom	MAE	0.1581	0.1429	0.1409	0.1329	0.1291
	(89,181)	(Training Time (s))	(6439)	(16382)	(6272)	(13797)	(8417)
	Band Gap	MAE	0.3576	0.3352	0.3339	0.3421	0.3207
	(89,181)	(Training Time (s))	(5051)	(24761)	(9203)	(8078)	(7660)
	Density	MAE	0.4100	0.3626	0.3617	0.3609	0.3333
	(89,181)	(Training Time (s))	(4783)	(19122)	(6659)	(9681)	(7330)
	E Above Hull	MAE	0.1166	0.1140	0.1205	0.1131	0.1098
	(89,181)	(Training Time (s))	(6904)	(22931)	(8625)	(9220)	(7985)
	Total Magnetization	MAE	1.4962	1.4637	1.4212	1.4222	1.4114
	(89,181)	(Training Time (s))	(4850)	(11990)	(5603)	(6159)	(5152)
JARVIS	Formation Energy/Atom	MAE	0.1261	0.1398	0.1037	0.1035	0.0925
	(19,994)	(Training Time (s))	(1505)	(4712)	(2109)	(3036)	(2350)
	Gap OPT	MAE	0.2960	0.3090	0.2840	0.2945	0.2679
	(17,924)	(Training Time (s))	(1502)	(4132)	(1669)	(2242)	(2137)
	Bulk Modulus	MAE	12.331	12.191	11.965	11.789	11.144
	(8,199)	(Training Time (s))	(553)	(3147)	(409)	(1593)	(981)
	Shear Modulus	MAE	11.010	10.580	10.535	10.392	10.043
	(8,199)	(Training Time (s))	(341)	(2056)	(450)	(1559)	(847)
	Gap TBMBJ	MAE	0.6194	0.5373	0.5663	0.5307	0.5270
	(5,287)	(Training Time (s))	(263)	(1710)	(671)	(1076)	(622)

From Table 5, we observe that our proposed model outperforms other DL models for all the datasets with different materials properties, which shows that irrespective of the materials representation that is used as the model input to train the DL models, the deep neural network with multiple callback functions significantly helps in accurately learning the materials properties as compared to other DL networks. We also see that the iBRNet is more accurate and requires less training time than its base architecture BRNet for almost all of the cases, which shows that the presence of multiple callback functions during the training phase of the neural network contributes towards producing a better model faster. Moreover, other pre-existing DL models that have less training time as compared to iBRNet have far worse test MAE than the proposed network making it not useful for further analysis. This shows the adaptability of the deep neural network with multiple callback functions for the general materials property predictive modeling task using any type of numerical vector-based representation as model input.

**Fig. 3:**
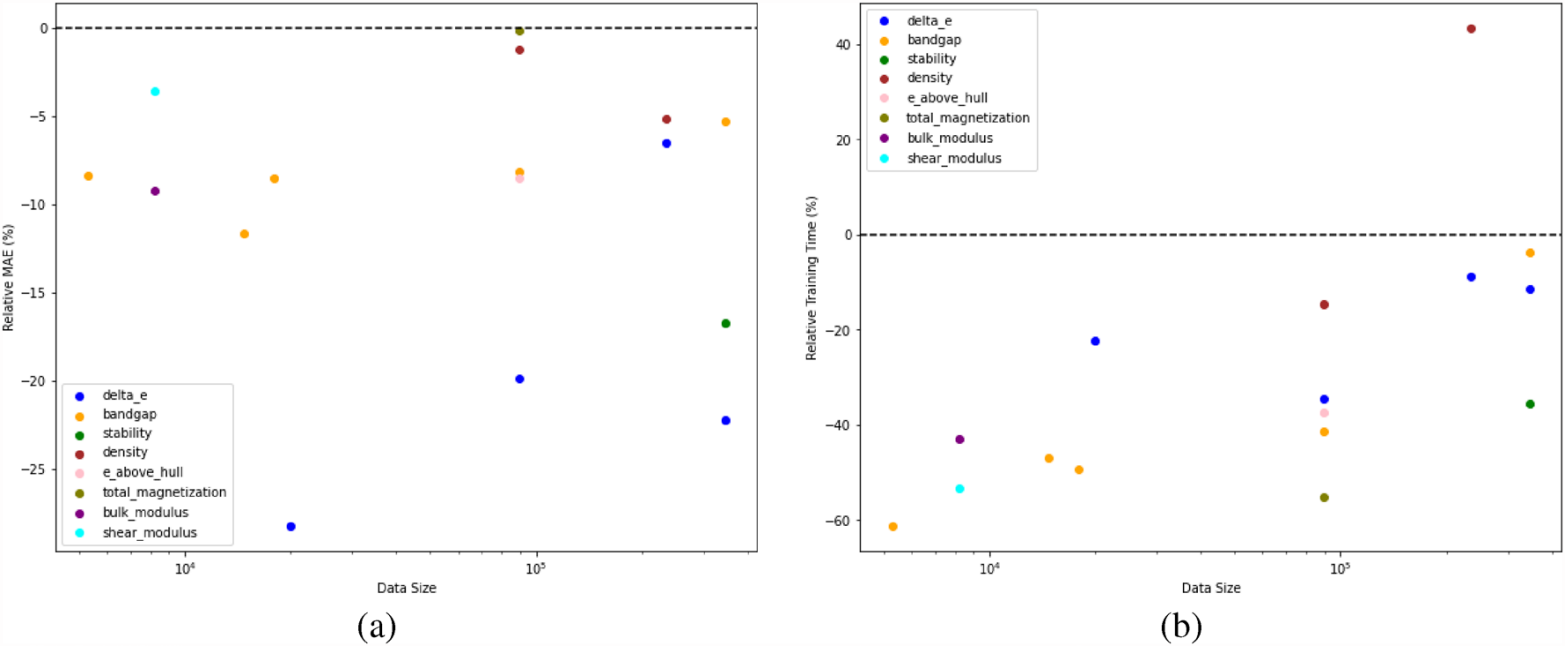
Impact of input representation on the accuracy and training time of iBRNet. The x-axis shows the dataset size on a log scale, and the y-axis shows the percentage change in: (**a**) test MAE and (**b**) training time of the model trained using composition-based elemental fraction as input w.r.t. the model trained using composition-based physical attributes as input (calculated as ((MAE_EF_/MAE_PA_)–1) x 100$\%$) for test MAE and (calculated as ((Time_EF_/Time_PA_)–1) x 100$\%$) for training time

Additionally, we investigate the impact of different composition-based input representations used for model training on the performance in terms of accuracy and training time of the model by comparing the elemental fraction (86 vector features representation) and physical attributes (145 vector features representation) using iBRNet in Fig. 3. In general, physical attributes are seen as a more powerful and informative set of descriptors as compared to elemental fractions. Interestingly, we observe that feature representation composed of elemental fractions performs better as compared to the physical attributes. We believe this might be due to the well-known deep neural network’s ability to work well on raw inputs without manual feature engineering [34, 53]. Hence, for further analysis, we will only use the feature representation composed of composition-based elemental fractions as model input.

### Comparison against other models

Finally, we investigate the performance of the proposed network against other well-known deep neural networks, i.e., Roost [48], CrabNet [49] and MODNet [50] that use composition-based features as model input in terms of MAE. We train iBRNet using feature representation composed of 86 vector composition-based elemental fractions as the model input. Roost uses matscholar [54] embedding comprised of composition and structure based information as input representations for graph neural networks (GNN). MODNet [50] featurizes composition based attributes from Matminer [52] and performs feature selection based on the specific materials property before feeding them into the neural network. CrabNet [49] uses mat2vec [54] embedding comprised of composition and structure based information as input representation for attention-based network.

**Table 6:** Test MAE of different models for each of the materials properties for prediction task of “Comparison against Other Models”

Dataset	Property (Size)	MODNet [55]	CrabNet [48]	Roost [49]	iBRNet
OQMD	Formation Energy/Atom (345,134)	0.0887	0.0506	0.0399	**0.0372**
	Band Gap (345,134)	0.0711	0.0668	0.0508	**0.0449**
	Stability (345,134)	0.0820	0.0443	0.0419	**0.0414**
AFLOWLIB	Formation Energy/Atom (234,299)	0.0699	0.0415	**0.0407**	0.0433
	Density (234,299)	0.2463	0.1770	0.1733	**0.1656**
	EGap (14,751)	0.1083	0.1410	0.1001	**0.0940**
MP	Formation Energy/Atom (89,181)	0.1605	0.1340	0.1061	**0.1035**
	Band Gap (89,181)	0.4674	0.3810	0.3413	**0.2943**
	Density (89,181)	0.4236	0.4320	0.3407	**0.3293**
	E Above Hull (89,181)	0.1324	0.1040	0.1015	**0.1005**
	Total Magnetization (89,181)	1.666	**0.1340**	1.3995	1.4094
JARVIS	Formation Energy/Atom (19,994)	0.1101	0.0926	0.0681	**0.0664**
	Gap OPT (17,924)	0.2959	0.3350	0.2713	**0.2451**
	Bulk Modulus (8,199)	13.276	10.600	11.072	**10.115**
	Shear Modulus (8,199)	12.696	10.100	10.012	**9.682**
	Gap TBMBJ (5,287)	0.5475	0.4870	0.4894	**0.4828**

Bold indicates the lowest MAE values

From Table 6, we observe that the proposed architecture outperforms the existing well-known deep neural network models in terms of test MAE for most of the cases, even though they comprise of complex architecture and informative input. This also shows the importance of hyperparameter selection and tuning for training deep neural networks. We believe this will inspire materials scientists to incorporate multiple schedulers for model training when building deep neural networks for the task of predicting materials properties.

### Performance analysis

Additionally, to visually illustrate the performance benefits of the proposed approach, we analyze the performance using a bubble chart, prediction error chart, and cumulative distribution function (CDF) of the prediction errors. In this analysis, we perform a comparative study of different deep neural networks comprised of the same number of layers in terms of the model accuracy and the training time using formation enthalpy of the four different DFT-computed datasets (OQMD, AFLOWLIB, MP, and JARVIS) as the materials property and composition-based elemental fractions as the model input.

**Fig. 4:**
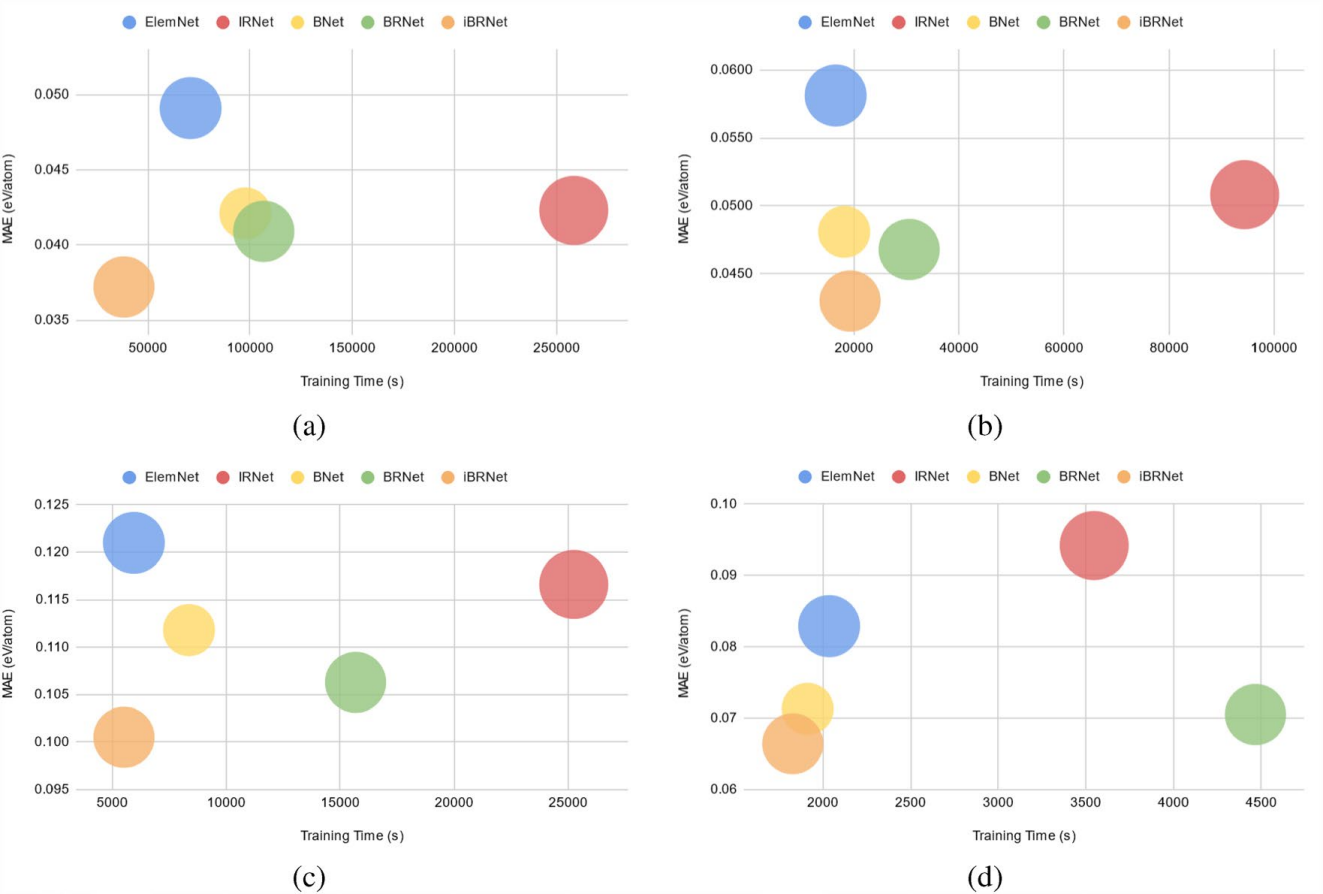
Bubble charts indicating the performance of the DL models based on the training time (s) on the x-axis, MAE (eV/atom) on the y-axis, and model parameters as the bubble size for (**a**) OQMD, (**b**) AFLOWLIB, (**c**) MP, and (**d**) JARVIS. The bubbles closer to the bottom-left corner of the chart are desirable as they correspond to less training time as well as low MAE

Figure 4 shows the bubble charts that indicate the performance in terms of training time on the x-axis, MAE on the y-axis, and bubble size as the model parameters for different DL models using formation energy as the materials property and composition-based elemental fractions as the model input. The bottom-left corner of the bubble chart corresponds to the better overall performance for a DL model, as it indicates that the approach can produce an accurate model with less training time. We observe the following trends from Fig. 4: 1. ElemNet and IRNet architectures that are constructed by stacking the layers components linearly and do not have multiple schedulers almost always perform poorly both in terms of accuracy and training time. Here, ElemNet is usually less accurate with faster training time, and IRNet is usually more accurate with slower training time; 2. BNet and BRNet architectures that are constructed by stacking the layers with branching and do not have multiple schedulers perform better as compared to ElemNet and IRNet in terms of accuracy and training time due to their architecture. Here, BNet is usually slightly faster in terms of training time, and BRNet is slightly better in terms of accuracy; 3. The proposed improved branched deep neural network architecture with multiple schedulers is always closest to the bottom-left corner of the bubble chart, showing that it is better as compared to other DL models without multiple schedulers in terms of model accuracy as well as training time when model training is performed in a controlled computational environment with parametric constraints.

**Fig. 5:**
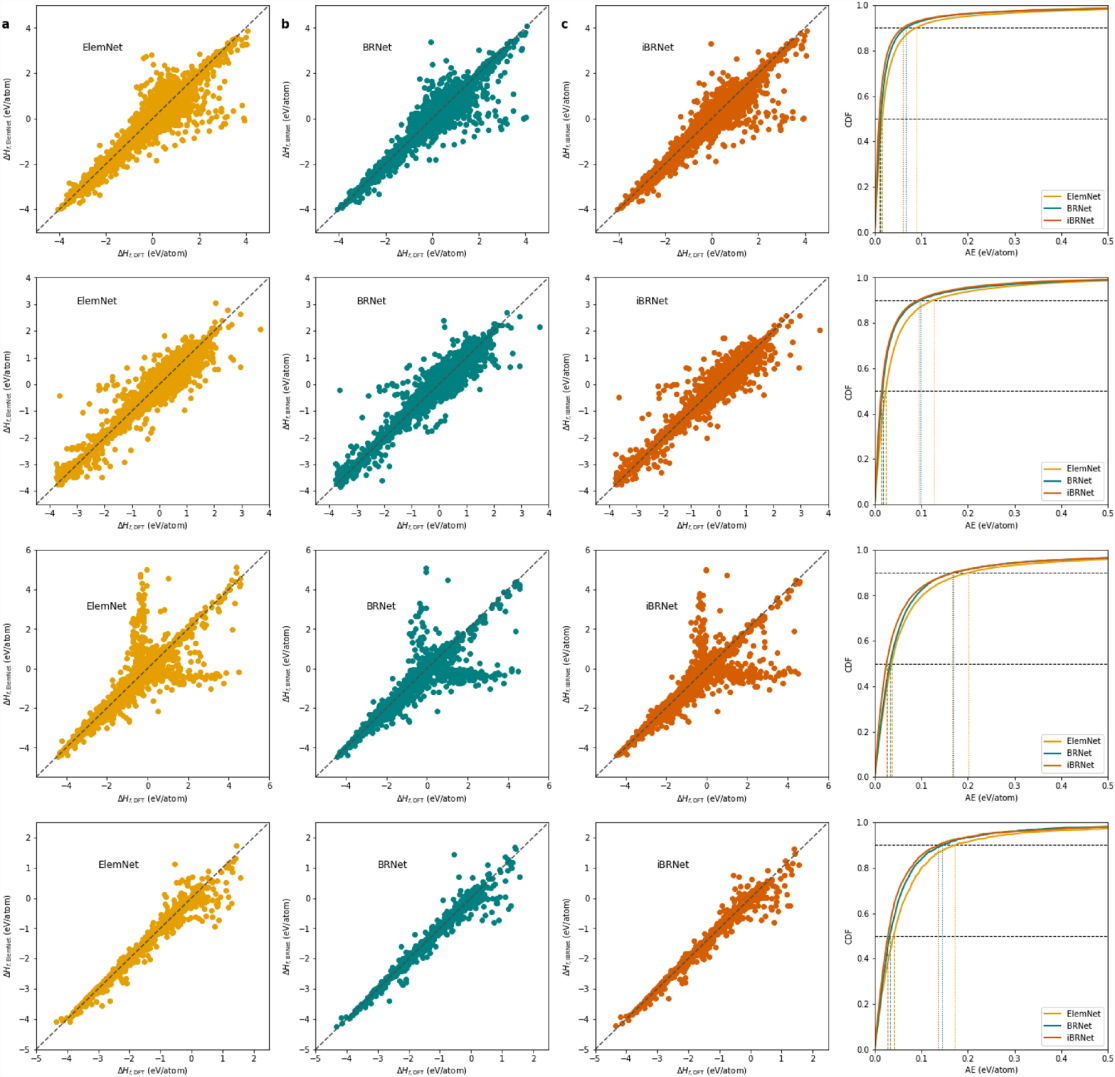
Comparison of ElemNet, BRNet against proposed iBRNet on formation energy as materials property and composition-based elemental fractions as model inputs. The rows represent different DFT-computed datasets in the order of OQMD, AFLOWLIB, MP, and JARVIS from top to bottom. Within each row, the first three subplots represent the prediction errors using three models: ElemNet, BRNet, and iBRNet; the last subplot contains the cumulative distribution function (CDF) of the prediction errors using the three models, with 50th and 90th percentiles marked

Figure 5 illustrates the prediction error chart and cumulative distribution function (CDF) of the prediction errors for formation energy as materials property and composition-based elemental fractions as model inputs using four DFT-computed datasets. Although we observe some similarity in the scatter plot of the ElemNet, BRNet, and iBRNet, the prediction and outliers for iBRNet are relatively closer to the diagonal for all the cases as compared to the other DL models. A few test points in Fig. 5 show a notable deviation between DFT-calculated and predicted energies. Such deviations usually stem from model/data bias caused by uneven coverage of materials classes in the dataset, as well as the differences in the materials property value distribution between train and test splits [56], and computational bias caused by parametric choices associated with DFT simulations to achieve reasonable accuracy across a wide variety of materials and properties [57]. Particularly, we observe two groups of large deviations, with horizontal deviation showing near-constant prediction values (which should exhibit different prediction values) and vertical deviation showing different prediction values (which should exhibit near-constant prediction values) in the MP dataset. In future work, it would be interesting to analyze what types of compounds fall into the area showing large deviations along with their underlying causes and implications. Moreover, comprehensive guides and practices to ensure standardization and interoperability among different simulation settings and diversity of materials classes and systems in datasets need to be ensured to mitigate such deviations. The CDF (cumulative distributive function) curves for the three models also help us better understand the difference in prediction error distributions, where for all four DFT-computed datasets, we observe lower 50th and the 90th percentile absolute prediction error for iBRNet as compared to ElemNet and BRNet. The bubble chart, prediction error chart, and cumulative distribution function (CDF) of the prediction errors demonstrate the advantage of incorporating multiple schedulers in a deep neural network for an improved overall predictive performance of the model trained in a controlled computational environment with parametric constraints.

## Conclusion

We presented a novel approach to incorporate multiple callback functions in deep neural networks to facilitate improved performance in terms of accuracy and training time for materials property prediction tasks in a controlled computational environment with parametric constraints. To demonstrate the advantages of the proposed approach, we built a deep neural network iBRNet, by using BRNet as the base architecture and introduce multiple callback functions during its model training. To compare the performance of the proposed model, we use existing deep neural networks which consist of the same number of layers in their architecture and do not incorporate multiple callback functions for their model training to ensure a fair comparison. The proposed model was first evaluated on the design problem of performing a predictive analysis on the formation energy of four different well-known DFT computed datasets. The proposed model significantly outperformed all the other existing deep neural networks in terms of accuracy and training time on the design problem. We also illustrate the generalizability of the proposed approach by comparing the performance of the proposed model with the existing well-known deep neural network, which comprises of complex architecture and informative input. Furthermore, we show the adaptability of the proposed model in terms of the input provided for model training by performing a predictive analysis of materials properties using different feature representations, i.e., composition-derived 86 vector elemental fractions and 145 vector physical attributes.

Overall, the proposed approach significantly outperforms other DL models in terms of accuracy and training time, irrespective of the data size and materials property being evaluated, where multiple callback functions demonstrate an effective and efficient ability to understand and analyze the hidden connection between a given input representation and the output property. Moreover, as our approach only requires little modification for the model training of the deep neural network, it does not affect the number of parameters required to build the deep neural network. But even with that small modification, we find that the proposed approach significantly reduced training time and even increased the accuracy of the model as compared to other baseline architectures used for comparison. Since the proposed approach of deep neural network with multiple callback functions is not dependent on any specific material representation/embedding to be used as model input for model training, it is expected to improve the performance of other DL works using other types of feature representations not only in materials science but other scientific domains as well. Combining the proposed approach with other innovations previously discussed, such as sophisticated networks and architectures, to evaluate its broad applicability would be an interesting future study. Interested readers can also explore different combinations of epochs for RLROP/ES to train the neural network or use more variety of callback functions in a bid to boost the performance of the target model for a specific materials property. The proposed approach of a deep neural network with multiple callback functions is conceptually simple to implement and build upon and is thus expected to be widely applicable. The iBRNet framework code is publicly available at https://github.com/GuptaVishu2002/iBRNet.

## Methods

The improved branched deep neural network architecture is created by using BRNet as the base architecture, which is formed by putting together a series of stacks, each composed of a fully connected layer and LeakyReLU [47] (except for the final layer, which has no activation function) with a branched structure in the initial layers and residual connections after each stack for better convergence during the training. The concept of branching and residual connection makes the regression learning task easier and provides a smooth flow of gradients between layers. “early stopping” and “reduce learning rate on plateau” were added as schedulers in this work for the multiple scheduler approach. The deep learning models were implemented using Python, TensorFlow 2 [58], and Keras [59]. Other hyperparameters for the deep neural networks were kept the same as the original work with Adam [60] as the optimizer, 32 as the mini-batch size, 0.001 as the (initial) learning rate, and mean absolute error as the loss function. For a detailed description of each of the deep neural networks, please refer to their respective publications [34, 40, 41, 61].

## Data Availability

All the datasets used in this paper are publicly available from their corresponding websites- OQMD (http://oqmd.org), AFLOWLIB (http://aflowlib.org), Materials Project (https://materialsproject.org), JARVIS (https://jarvis.nist.gov), and using Matminer (https://hackingmaterials.lbl.gov/matminer/). The simplified implementation of the proposed network used in this work is publicly available at https://github.com/GuptaVishu2002/iBRNet.
